# Male Reproductive Toxicity of Antifouling Chemicals: Insights into Oxidative Stress-Induced Infertility and Molecular Mechanisms of Zinc Pyrithione (ZPT)

**DOI:** 10.3390/antiox13020173

**Published:** 2024-01-29

**Authors:** Mogellah John Panga, Ye Zhao

**Affiliations:** School of Pharmaceutical Sciences, Nanjing Tech University, Nanjing 211816, China

**Keywords:** antifouling chemicals, ZPT, oxidative stress, male reproductive toxicity, sperm

## Abstract

Zinc pyrithione (ZPT), a widely utilized industrial chemical, is recognized for its versatile properties, including antimicrobial, antibacterial, antifungal, and antifouling activities. Despite its widespread use, recent research has shed light on its toxicity, particularly towards the male reproductive system. While investigations into ZPT’s impact on male reproduction have been conducted, most of the attention has been directed towards marine organisms. Notably, ZPT has been identified as a catalyst for oxidative stress, contributing to various indicators of male infertility, such as a reduced sperm count, impaired sperm motility, diminished testosterone levels, apoptosis, and degenerative changes in the testicular tissue. Furthermore, discussions surrounding ZPT’s effects on DNA and cellular structures have emerged. Despite the abundance of information regarding reproductive toxicity, the molecular mechanisms underlying ZPT’s detrimental effects on the male reproductive system remain poorly understood. This review focuses specifically on ZPT, delving into its reported toxicity on male reproduction, while also addressing the broader context by discussing other antifouling chemicals, and emphasizing the need for further exploration into its molecular mechanisms.

## 1. Introduction

Antifouling agents play a crucial role in chemical manufacturing facilities by ensuring the optimal operation and high productivity of chemical reactors. Strategies to combat fouling are employed across maritime, medical, and industrial settings [1]. Various techniques such as applying hydrophobic polymer coatings containing nitrofurazone, using hydrophobic synthetic self-cleaning paints, and employing silver-based hydrogels are utilized to mitigate various forms of fouling [2,3]. The ideal antifouling agent should possess desirable properties while minimizing potential harm to marine life in close proximity to the submerged surfaces [1]. However, it is worth noting that certain antifouling chemicals such as triphenyltin (TPT), tributyltin (TBT), zinc pyrithione (ZPT), copper, and cadmium have been documented to exhibit toxic effects [4,5,6,7,8,9,10].

The term “reproductive toxicity” encompasses the detrimental effects of a substance on any stage of the reproductive cycle, including impacts on the fetus or offspring as well as the impairment of reproductive function in both male and female organisms [11]. Specifically, male reproductive toxicity refers to the intrusion of harmful substances into the male reproductive system, resulting in disruptions in normal functioning. Typically, this leads to harm to the sperm and testicles. Anomalies in semen parameters such as a reduced sperm motility, density, viability, and count, along with increased DNA damage and abnormal morphology, can be attributed to testicular and sperm damage. In addition to genetic disorders, exposure to exogenous chemicals, including endocrine disruptors and medications commonly used in chemotherapy, is a prevalent cause of abnormal male reproduction in humans [12,13]. Chemical exposure adversely affects testicular cells and the hormonal environment, ultimately leading to testicular dysfunction and diminished semen quality, thereby reducing male fertility [14,15]. Male reproductive toxicity has been a persistent concern, with environmental chemicals being identified as one of the contributing factors [11]. The decline in male fertility worldwide has been linked to various environmental influences. To comprehend the adverse effects of these chemicals, it is beneficial to begin by examining their distinct properties. Arzuaga X. et al. have extensively characterized male reproductive toxicants, highlighting several significant attributes including genotoxicity, oxidative stress, perturbations in reproductive hormone levels and production, DNA damage, disruptions in germ cell development and viability, inflammation, and potential epigenetic alterations [16].

**Figure 1:**
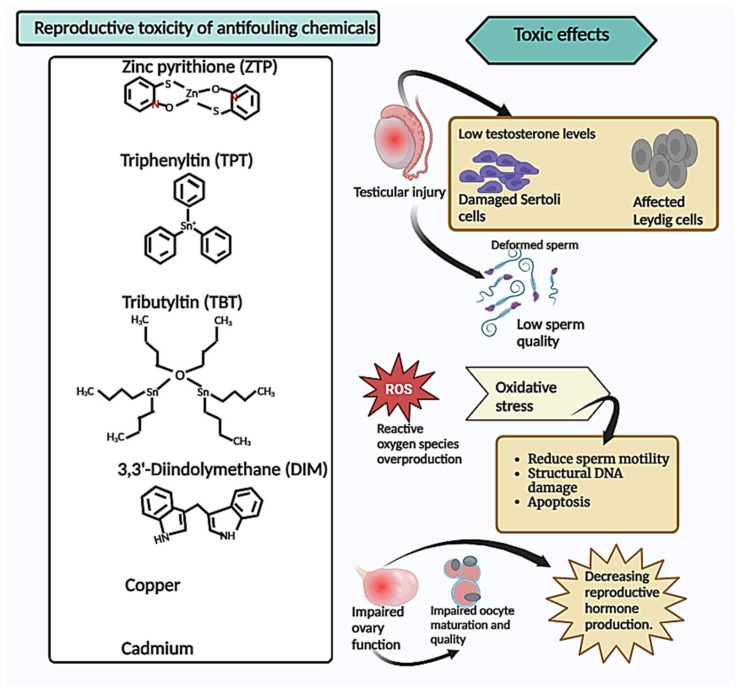
Reproductive toxicity of antifouling chemicals. Summary of the toxic effects of antifouling chemicals on the reproductive system, indicating that they cause testicular injury leading to a decrease in testosterone and cell damage, which causes sperm deformities and low sperm quality and quantity. Additionally, these chemicals induce oxidative stress, resulting in reduced sperm motility, structural DNA damage, and apoptosis. Furthermore, these antifouling chemicals impair ovary function, leading to impaired oocyte maturation and a decrease in reproductive hormone production.

Environmental pollutants can have detrimental effects on the reproductive systems of organisms, persisting for up to four generations following in utero exposure. These effects can be attributed to alterations in the gene expression associated with spermatogenesis and steroidogenesis, disruptions in the endocrine system (acting as endocrine disruptors), and epigenetic modifications [12,17,18]. Numerous studies have documented the adverse effects of antifouling chemicals on the reproductive system, as depicted in Figure 1 [6,7,9,19]. The available data indicate that the organotin compound TPT is known to induce reproductive toxicity, disrupt endocrine function, decrease testosterone production, and generate reactive oxygen species in both aquatic and mammalian species [20,21,22,23]. TBT is an additional antifouling chemical known for its reproductive toxicity, as it has the potential to disrupt spermatogenesis and alter fish behavior, thereby affecting male reproduction in zebrafish [24,25]. Empirical studies have provided evidence of a positive association between cadmium and copper exposure and reproductive toxicity [26,27,28,29]. Cadmium (Cd) has long been the subject of extensive scientific investigation as an environmental contaminant with detrimental impacts on the reproductive system [30,31,32]. Recent research suggests that Cd exposure may adversely affect the male reproductive system through mechanisms such as oxidative stress, inflammation, the disruption of steroidogenesis, and epigenetic modifications [33,34]. The study conducted by Cupertino M.C. et al. on male Wistar rats exposed to Cd revealed that Cd-mediated toxicity is associated with damage to the testes, both morphologically and functionally, resulting in decreased testosterone levels [35]. Numerous studies have been conducted to investigate the reproductive toxicity of copper, revealing its detrimental effects on testicles, induction of ovary toxicity, reduction in sex hormone secretion, elevation of oxidative stress levels, acceleration of apoptosis, deterioration of sperm quality, and increased incidence of malformations [36,37,38]. Environmental exposure to copper may have adverse consequences on spermatozoa apoptosis, impaired sperm DNA integrity, and compromised semen quality in humans [39]. In Nile tilapia fish (*Oreochromis niloticus*), copper has been demonstrated to exert toxicity on the male reproductive system, leading to significant declines in progressive motility percentages, motile ratios, sperm concentrations, and testicular testosterone levels [40,41]. Oriakpono O.E. et al. investigated the impact of petrol and cuprous oxide on albino rats (*Rattus norvegicus*) by assessing specific parameters related to rat semen and hematology. Their findings revealed that cuprous oxide exerts detrimental effects on the male reproductive system [42]. ZPT has been observed to induce alterations in oxidative stress levels and disrupt metal homeostasis in both human cell models and aquatic organisms [43,44]. Additionally, it can impede membrane transport, interfere with the synthesis of cellular ATP, and form complexes with proteins and metals within the cellular environment [45]. Given the findings, it is crucial to gain a comprehensive understanding of the reproductive toxic effects exhibited by ZPT and the other antifouling agents listed, as well as the underlying mechanisms responsible for these effects.

## 2. Male Reproductive Effects of Antifouling Agents

Antifouling chemicals have been found to possess a significant toxicity towards the male reproductive system. ZPT, a complex comprising of a pair of pyrithione rings connected to the central metal ion through a bridge formed by zinc and oxygen atoms, exhibits antimicrobial properties and demonstrates a broad spectrum of antibacterial activity against various microorganisms, including bacteria, fungi, and both gram-positive and gram-negative strains [46,47,48,49]. Notably, its extremely low solubility allows for its formulation and application as a particulate material, offering distinct performance advantages [47]. ZPT has been widely utilized in the field of dermatology for over five decades, primarily due to its effectiveness in treating conditions such as dandruff and seborrheic dermatitis [46,49,50]. Its application has since expanded to include skincare products. In the industrial sector, ZPT has been incorporated as an antifouling agent in aquatic adhesives since the 1990s [51,52]. Despite the long-standing use of ZPT as a compound with established safety, it has been suggested that metal pyrithiones, including ZPT, may persist in low-light marine environments [44,53,54], such as heavily turbid water columns or areas shaded beneath stationary boats in marinas and harbors [55]. This persistence can lead to the accumulation of ZPT, potentially resulting in adverse effects [56]. Furthermore, research indicates that the breakdown products of zinc pyrithione in the presence of light may impede fish cholinesterase enzyme activity, thereby posing a risk to fish nervous systems [43,44]. Numerous studies have provided evidence of the inhibitory impact of zinc pyrithione (ZPT) on membrane transport, cellular ATP synthesis disruption, and the formation of complexes with metals and proteins within the cellular environment. This is further elucidated in a comprehensive investigation examining the consequences on the survival and reproductive capabilities of the marine polychaete *Dinophilus gyrociliatus* [57]. Notably, ecologically relevant concentrations of ZPT have been found to exert deleterious effects on crucial traits, including oxidative and neurotoxic effects, as well as modifications in the swimming patterns of *Daphnia longispina* and *magna* [58]. Furthermore, ZPT has been associated with several adverse effects, such as the induction of apoptosis, impairment of the immune response, and alteration of the compositions of affected plant communities [59].

It is important to acknowledge that ZPT is widely present in the environment, which exposes humans to potential exposure through various routes, including dietary sources and dermal absorption. Holmes A.M. et al. conducted a study demonstrating that the topical application of ZPT formulations increases the absorption of zinc by human skin, thereby raising concerns about localized toxicity [60]. In a toxicity and accumulation study involving Carassius sp., one of the most extensively cultivated and distributed fish species in China, it was observed that ZPT can accumulate in tissues such as the liver and kidney, exhibiting a prolonged tissue residual time [61]. Extensive research has provided insights into the reproductive toxicity of ZPT, particularly in male aquatic species. Sperm motility, a crucial factor in male fertility, is influenced by temperature, osmolality, ATP concentration, and ion concentrations. Furthermore, assessing sperm quality relies heavily on sperm motility [62]. Previous investigations have elucidated that exposure to ZPT significantly diminishes the motility and alters the motion patterns of human spermatozoa, thereby impeding the normal process of sperm cell maturation and compromising their reproductive capability [43]. Given that the secretion of testosterone is crucial for spermatogenesis, a decline in testosterone levels can conceivably correlate with an escalation in the frequency of sperm abnormalities as well as a deterioration in the quality of spermatozoa [63,64]. Pertinently, ZPT has been extensively reported to induce a remarkable reduction in testosterone levels, instigate the disruption of acrosomal membrane integrity, give rise to deformities within the spermatozoa, which unequivocally diminish their quality and quantity, and substantially impair the overall sperm function in zebrafish samples [65].

The utilization of the organotin compound TPT is observed in plastic stabilization and agriculture, owing to its insecticide and fungicide properties [21]. Moreover, it is extensively employed in the production of antifouling paints for ships on a global scale [21]. TPT has been associated with the formation of malondialdehyde, oxidative stress, and the activation of antioxidant defense enzymes such as superoxide dismutase and catalase. Within the framework of ecotoxicological investigation, it has been extensively employed as a reliable biomarker of oxidative stress [9]. Earlier investigations have delved into the male reproductive toxicity of this compound, and it was reported that by disturbing the microenvironments of Leydig and Sertoli cells, TPT diminishes the proliferative potential of spermatogonia, leading to the production of subpar sperm in adult male rats [66]. Furthermore, exposure to TPT during pregnancy exerts an impact on the development of the fetal testis in rats, resulting in a dose-dependent reduction in serum testosterone levels, the induction of fetal Leydig cell aggregation, and a decrease in the size of the fetal Leydig cells [67]. TBT, an artificial organic derivative of tin, exhibits a chemical structure in which covalent bonds are formed between carbon and tin atoms. Its primary application revolves around its utilization as a biocide, effectively preserving paper, wood, textiles, leather, and industrial water systems [68,69]. Moreover, TBT plays a pivotal role as a stabilizer during the manufacturing process of plastic goods. However, due to the deleterious impacts it poses on marine ecosystems, the domestic and global regulatory bodies have introduced comprehensive bans on the usage of TBT. Despite these regulations, TBT displays a remarkable persistence in the environment and tends to bioaccumulate, thereby infiltrating the food chain, including through dairy products, meat, fish, and even the human body [68,69]. Intriguingly, Mitra et al. conducted an in vitro investigation reporting TBT to function as an endocrine disruptor, specifically influencing testicular cells in male Wistar rats, which could potentially lead to male infertility [70]. Notably, the testosterone levels were found to diminish, spermatogenesis faced impairments, antioxidants were affected, and DNA damage responses were induced in the spermatozoa of the freshwater prawn *Macrobrachium rosenbergii* [71,72,73]. This notion is reinforced by the research findings of Lan et al., in which TBT was observed to cause a decline in the sperm count and testis weight, initiate apoptosis, and negatively regulate the gene expressions responsible for germ cell proliferation (namely cyclin d1 and PCNA) in zebrafish [24]. For a summary overview of antifouling agents, their effects on sperm quality, and the specific species targeted, refer to Table 1 below. 

## 3. Role of Antifouling Agents in Regulating Oxidative Stress-Induced Sperm and Testicular Injury

Extensive research has been conducted to investigate the pivotal role played by oxidative stress in determining sperm quality [74,75,76,77,78]. Redox homeostasis, despite the potential misnomer associated with its name, represents an intricately dynamic process whereby the stability of the redox status within cells is meticulously safeguarded by an exquisitely responsive system that promptly detects alterations in the redox status and acutely readjusts metabolic activities to restore the optimal redox balance [79]. Notwithstanding the presence of defense mechanisms, such as proteins, enzymes, and vitamins aimed at combating free radicals, the accumulation of reactive oxygen species (ROS) can still transpire due to the malfunctioning or excessive production of the antioxidant system, thereby leading to the incipient phase characterized as “oxidative stress” [80,81]. The state of oxidative stress within a cellular entity is not solely characterized by an excessive accumulation of ROS, but also by a conspicuous decline in the antioxidative potential exhibited by said cell. It is worth noting that ROS, due to the presence of unpaired electrons, exhibit a heightened reactivity and act as potent oxidizing agents [82]. At physiologically relevant concentrations, ROS notably contribute to the acquisition of pivotal properties deemed necessary for sperm fertilization, including but not limited to motility facilitation, chemotaxis regulation, promotion of sperm capacitation, induction of hyperactivation, initiation of the acrosome reaction, orchestration of oocyte interactions, and even compaction of the chromatin structure within maturing spermatozoa [78]. This assemblage of events collectively represents the indispensable influence of ROS on sperm functionality, albeit the excessive production of ROS stands as a prominent causative agent for sperm deterioration. In fact, spermatozoa are unenviably susceptible to oxidative damage owing to their disproportionately high concentration of membrane-based unsaturated fatty acids compounded by the lack of cytoplasmic antioxidant enzymes, both of which synergistically contribute to the observed adverse impact on sperm quality and overall functionality [76,83,84].

Numerous intricate mechanisms are in place to regulate the generation and elimination of ROS to maintain optimal ROS levels required for physiological processes. These mechanisms encompass localized ROS generation or redox compartmentalization, as well as the involvement of cofactors and antioxidant systems that effectively detoxify ROS [85]. It is important to note that ROS generation represents only a fraction of the overall redox regulation involved in sperm function. Male gametes exhibit a distinctive pattern of antioxidant enzymes compared to other cellular or organ systems, characterized by a relatively higher concentration of glutathione peroxidase (GPx) and superoxide dismutase (SOD), while possessing a comparatively lower amount of catalase (CAT) [86]. SOD, being the primary antioxidant enzyme responsible for ROS detoxification, effectively reduces the potentially hazardous superoxide anions by catalyzing the conversion of two anion molecules into hydrogen peroxide and molecular oxygen [87]. Subsequently, the breakdown of hydrogen peroxide into water and molecular oxygen occurs through the action of CAT, which serves as another vital antioxidant enzyme, completing the detoxification cycle initially mediated by SOD [87,88]. Remarkably, glutathione peroxidase (GSH-Px) operates as an indispensable nonenzymatic antioxidant within the mitochondria and cytosol, safeguarding cells against oxidative stress by catalyzing the conversion of lipid peroxides into alcohols and hydrogen peroxides into water [87,89]. ROS-induced oxidative stress exerts myriad effects on the parameters of sperm. The detrimental impact of oxidative stress on sperm includes the induction of cell death pathways and disruption of sperm production, culminating in a decremented sperm count, oligozoospermia, augmented apoptosis, and impaired sperm viability [90,91]. Additionally, oxidative stress hampers the forward motility of sperm (sperm motility) by impeding flagellar movement, perturbing the integrity and fluidity of the sperm plasma membrane, and modifying the membrane composition [92,93]. It is noteworthy that ROS-induced oxidative stress precipitates direct impairment in the structural components of the sperm, such as the plasma membrane, mitochondria, and DNA, consequently leading to aberrant morphological alterations [94,95].

Notably, antifouling agents such as ZPT, TBT, and TPT have been implicated in regulating the generation of ROS and modulating antioxidant enzyme activities, thereby contributing to the intricate molecular mechanisms that govern redox homeostasis [96,97,98]. The uncontrolled generation of ROS precipitates a state of oxidative stress, exerting profound effects on lipid peroxidation, DNA fragmentation, and mitochondrial function with the subsequent induction of apoptosis, compromised sperm morphology, and impaired sperm functionality [76]. The highly condensed and tightly packed nature of sperm cell DNA renders it particularly vulnerable to oxidative damage instigated by ROS. The deleterious consequences of oxidative stress, encompassing DNA breakage and oxidative modifications, impede the sperm’s capacity to successfully fertilize eggs, thereby compromising its genetic soundness [99]. In addition to triggering DNA fragmentation, oxidative stress carries the potential to give rise to chromosomal abnormalities and genetic mutations within sperm [100]. The fragmentation of DNA provokes apoptotic mechanisms and markedly influences the sperm count [101,102]. Instances of oxidative stress-induced DNA damage in sperm cells have been ascribed to antifouling agents, where high doses of TBT have been evidenced to induce DNA damage in the prawn species *Macrobrachium rosenbergii* [71]. In zebrafish (*Danio rerio*), the induction of apoptosis by TBT has been evidenced. This induction is linked to an augmentation in the population of spermatocytes that exhibit an apoptotic activity [24]. Moreover, TBT has also been proven to suppress the mRNA levels of the gene encoding the terminus of meiotic entry (*cyp26a1*), while concurrently upregulating the expressions of genes involved in meiotic entry and maintenance (*aldhla2*, *sycp3*, and *dmc1*) [24]. In contrast, the expressions of genes responsible for germ cell proliferation (*cyclind1* and *pcna*) were found to be downregulated [24]. These findings consistently support the notion of disrupted meiosis and a reduced sperm count. Furthermore, the impact of TBT extends to the modulation of gene expression involved in the intrinsic and extrinsic apoptotic pathways (*bax/bcl-2* and *tnfrsf1a/tnfrsf1b*) within the testes of zebrafish [24]. Another study by Wang Y.X. et al. has indicated that copper exerts detrimental effects on the DNA of sperm tails, consequently leading to a decreased semen quality [39]. Additionally, ZPT has been implicated in the induction of ROS-mediated DNA damage in testicular tissue of zebrafish [65].

The mitochondrion, an organelle present in spermatozoa, assumes the role of generating ROS. It is worthy to note that the electron transport chain situated on the mitochondrial membrane can be influenced and modified by diverse factors encompassing electromagnetic radiation, polyunsaturated fatty acids, and apoptotic elements, ultimately resulting in an excessive generation of ROS [103]. Through the process of oxidative phosphorylation, mitochondria serve as a fundamental entity for generating energy and synthesizing adenosine triphosphate (ATP), which in turn, plays a crucial role in ensuring the motility and viability of sperm cells [104]. The proper functioning of mitochondria is indispensable as their malfunctioning can impede the maintenance of sperm vitality and viability. In fact, oxidative stress-induced mitochondrial dysfunction can exert a deleterious impact on sperm cells by intensifying apoptotic signaling and triggering programmed cell death [105]. Over time, certain substances used to prevent fouling have been found to have negative effects on the proper functioning of mitochondria. This was demonstrated in a scientific investigation conducted on the species *Calomys laucha*, which emphasized the harmful impacts of exposure to TPT on various cellular components, including a decrease in mitochondrial function. As a result, this reduction ultimately leads to a decline in the overall activity and quality of sperm cells [21]. Daigneault B.W. et al. presented an investigation that explored the consequences of subjecting frozen–thawed bovine spermatozoa to TBT, revealing a noteworthy decline in the sperm mitochondrial membrane potential and a consequent reduction in sperm motility [106]. Moreover, the research findings indicated a robust and significant impact of ZPT on the depolarization of human spermatozoal mitochondria, ultimately leading to the immobilization of spermatozoa [43]. Furthermore, by inducing the activation of caspase 3 and caspase 9 as well as modulating the Bcl2a/Bax ratio, ZPT was observed to amplify mitochondrial apoptosis [107,108]. In addition to this, a myriad of studies has consistently demonstrated that ZPT instigates apoptosis in various cell types, including but not limited to HepG2 cells, mouse thymocytes, and lymphocytes, through its interference with mitochondrial functionality [108,109]. ROS initiate lipid peroxidation, comprising a cascade of chemical reactions that selectively target the polyunsaturated fatty acids present in the cellular membranes of spermatozoa. This biological process gives rise to lipid peroxides, which disrupt the structural integrity of the cell membrane. Consequently, the functionality and viability of the sperm are compromised, resulting in a diminished motility [110]. The foremost line of defense against such oxidative damage is provided by endogenous antioxidants, primarily encompassing GPx, CAT, and SOD. These antioxidants play a pivotal and indispensable role in the overarching strategy employed by the organism to counteract oxidative stress [87]. Nevertheless, it is worth noting that they can be influenced by antifouling chemicals, thereby subject to regulation. TBT has been reported to induce a significant reduction in the levels of GPx and SOD antioxidants within the prawn species *Macrobrachium rosenbergii*, adversely affecting sperm protection, activation, and various functional attributes [71]. Additionally, it has been demonstrated that the antifouling agent ZPT modulates the abundance of GPx, CAT, and SOD, consequently provoking oxidative stress in the spermatozoa of zebrafish [65].

It has been demonstrated that the buildup of ROS in the endoplasmic reticulum (ER) during stress exacerbates oxidative stress. Similarly, oxidative stress can worsen ER stress by disrupting protein folding pathways [111,112]. The imbalance in the redox state plays a role in the activation of autophagy during ER stress. This is supported by the expression of marker genes related to autophagy in zebrafish embryos treated with ZPT [113]. The treatment with ZPT leads to a significant increase in the expression of autophagy-related genes 5 (*atg5*) and 6 (*atg6*, also known as *beclin1*) in various concentrations. Autophagy-related 16 like 2 (*atg16l2*) is also found to be increased under these conditions [113,114]. In moderation, ROS are essential for regulating several intracellular signaling pathways, the immune system, mitogen responses, and cellular homeostasis [115]. ROS induces the production of cyclic adenosine monophosphate (cAMP) in sperm, leading to tyrosine phosphorylation through the inhibition of tyrosine phosphatase. This molecular mechanism activates numerous transcription factors involved in intracellular signaling cascades for sperm physiology [86]. ROS also facilitates capacitation by elevating intracellular cAMP levels and triggering the activation of protein kinase A (PKA) [116,117]. Treatment with ZPT in human spermatozoa results in a H^+^ accumulation and Ca^2+^ dissipation, along with the suppression of the cAMP/PKA signaling pathway, leading to the immobilization of spermatozoa [43]. The inhibition of spermatogenesis in the lined seahorse (Hippocampus erectus) can occur due to exposure to TBT, which suppresses the cyclic AMP activity [118]. The data suggest that these specific pathways may be regulated by substances possessing antifouling properties.

Given the existing body of knowledge concerning the control of testicular harm and sperm affected by antifouling substances, it would be beneficial to delve into additional research regarding the intricate mechanisms that govern redox homeostasis and how these antifouling agents affect or govern it. Another crucial realm necessitating further exploration is comprehending the repercussions of ZPT and other antifouling compounds on intracellular signaling pathways that play a pivotal role in the male reproductive system.

## 4. Molecular Mechanisms of ZPT in Oxidative Stress-Induced Male Reproductive Toxic Regulation

**Figure 2:**
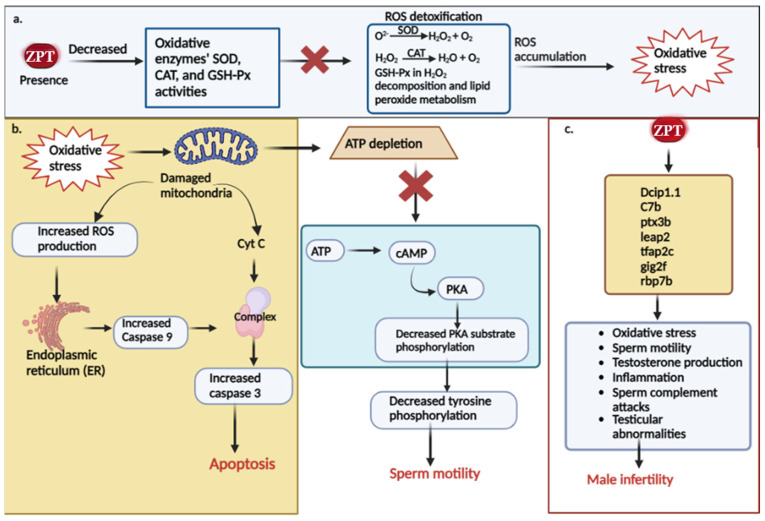
Molecular mechanisms of ZPT in male reproductive toxic regulation. (**a**) ZPT’s effects on oxidative parameters, overproduction of ROS, and induction of oxidative stress. (**b**) Mechanism of ZPT-induced oxidative stress in causing apoptosis and affecting sperm motility. (**c**) The impact of ZPT on the regulation and expression of reproductive genes and how it affects male fertility.

Up to this point, several researchers have presented findings on the toxicological mechanisms of ZPT on male reproductive function. Figure 2 provides a concise summary of these mechanisms. Numerous studies have indicated that ZPT can induce the excessive production of ROS, leading to the development of oxidative stress [58,96,119,120]. As illustrated in Figure 2a, the impact of ZPT on male reproductive toxicity involves interference with ROS signaling mediation by promoting ROS overproduction. Consequently, this disturbance can lead to alterations in oxidative stress markers by reducing the presence of crucial enzymes such as GSH-Px, SOD, and CAT. These enzymes play a pivotal role in neutralizing ROS through various chemical processes, including the breakdown of hydrogen peroxide and lipid peroxide metabolism [65]. Any disruption in these processes ultimately leads to the development of oxidative stress.

Moreover, the excessive production of ROS can lead to oxidative damage of the mitochondria, which are vital for controlling apoptosis, ROS levels, and ATP synthesis [43,121]. It is widely recognized that the process of mitochondrial apoptosis initiates with the depolarization of the mitochondrial membrane, resulting in the loss of membrane potential, the disruption of oxidative phosphorylation, an amplified generation of reactive oxygen species, and the diffusion of mitochondrial components into the cytosol, of which among these components is cytochrome c [122]. Upon its release into the cytosol, cytochrome c forms the apoptosome, which activates caspase 3, the major executor, in response to apoptotic signals. Additionally, it prompts the binding of nucleotides to Apaf-1, thus promoting caspase 9 activation [123]. Studies have shown that ZPT enhances the activities of caspase 3 and caspase 9, indicating its ability to induce the caspase 3- and caspase 9-dependent mitochondrial apoptotic process [113]. Furthermore, ER stress can initiate apoptotic pathways to eliminate damaged cells [124]. The oxidative stress induced by ZPT can induce ER stress, leading to caspase 3 activation and subsequent apoptosis [113]. It has been determined that PKA acts as a key regulator, functioning as the downstream effector of the cAMP/PKA signaling pathway and aiding in the regulation of spermatozoa motility and male fertility [125]. Notably, ZPT significantly reduces the phosphorylation levels of PKA substrates and tyrosine residues in human spermatozoa, suggesting that the ZPT-mediated immobilization of spermatozoa involves the cAMP/PKA pathway [43]. Figure 2b visually demonstrates how ZPT-induced oxidative stress interferes in the regulation of these mechanisms.

Male infertility is caused by several gene biomarkers. One such biomarker is *Dcip1.1* in zebrafish, which is believed to be involved in T activation and has been associated with oligospermia [126]. Another gene, *C7b*, is connected to male infertility, oligospermia, and testicular diseases. It is predicted to function upstream or within complement activation [127,128]. *Ptx3b*, on the other hand, is expected to play a role in the inflammatory response by facilitating the binding activity of the C1q complex, which protects sperm from complement attacks [129]. *Leap2* has also been linked to male infertility, specifically, low sperm counts and testicular abnormalities. It is thought to function upstream or within the defensive response to bacteria [130,131]. *Tfap2c* controls TEAD4 transcription, enhancing the activation of Th17 and Th1 cells, which in turn leads to systemic inflammation. This inflammation can reduce testosterone levels, alter the morphology, and reduce sperm motility, thereby impacting male fertility [132,133]. Another important gene, *gig2f*, is expected to increase the activity of NAD+ADP ribosyl transferase in zebrafish. The overexertion of this enzyme can damage testicular function and inhibit sperm motility, contributing to oxidative stress and male infertility [134]. The *rbp7b* gene is associated with the transport of fatty acids and promotes a fatty acid-binding activity when expressed. Trans fatty acids have been found to accumulate in the testes of male mice, resulting in reduced serum testosterone levels, sperm counts, spermatogenesis inhibition, and testicular degeneration [135,136]. Hu et al.’ s research demonstrates that ZPT can regulate and control the expression of these gene biomarkers in zebrafish [65] (Figure 2c).

Researchers have proposed potential molecular mechanisms for the regulation of male reproductive toxicity by ZPT [43,65]. Nevertheless, the available information falls short in providing a comprehensive understanding of the specific pathways involved in this regulation. To gain deeper insights, it is imperative for researchers to concentrate on future investigations regarding the effects of ZPT-induced oxidative stress on signaling pathways, leading to apoptosis, sperm motility impairment, DNA damage, decreased testosterone levels, and the inhibition of spermatogenesis. Furthermore, unveiling the precise mechanism through which ZPT controls the regulation and expression of male fertility gene biomarkers is another aspect that researchers can explore to advance our comprehension in this field.

## 5. Conclusions

As explored in the comprehensive analysis, oxidative damage to cells may have a detrimental effect on male fertility. This damage has the potential to disturb the lipids present in sperm mitochondrial membranes and hinder specific protein kinases. Moreover, it can elevate the level of damage sustained by sperm DNA, leading to a decrease in the sperm motility, count, and overall function, influencing the sperm morphology as well. It is widely acknowledged that an impaired regulation of the molecular mechanisms responsible for ROS production engenders oxidative stress. Consequently, we have examined the influence of antifouling agents, namely ZPT, TBT, and TPT, on ROS production regulation as well as their impact on important antioxidant enzymes, SOD, CAT, and GPx, which participate in the detoxification of ROS. Gaining a comprehensive understanding of the mechanisms underlying the male reproductive toxicity induced by ZPT and other antifouling compounds can aid in developing a more targeted and efficient screening approach for these chemicals. Additionally, this knowledge may enable the prioritization of chemicals with limited data for further evaluation and assessment.

## Figures and Tables

**Table 1:** Antifouling agents’ effects on sperm quality with specified species.

Antifouling Chemical	Effect on Sperm Quality	Species	References
Zinc pyrithione (ZPT)	Decreases motility	Frozen–thawed human sperm	[43]
	Decreases testosterone levels, increases abnormalities, decreases quantity, and lowers quality	Zebrafish (*Danio rerio*)	[65]
Tributyltin (TBT)	Lowers testosterone levels, damages DNA, and affects antioxidants	Freshwater prawn (*Macrobrachium rosenbergii*)	[71,72]
	Lowers sperm count and induces apoptosis	Zebrafish (*Danio rerio*)	[24]
Triphenyltin (TPT)	Reduces sperm count and motility, increases deformities, and upsets the Leydig and Sertoli cell microenvironments	Sprague Dawley rats	[66]
Cadmium	Lowers testosterone levels and causes DNA fragmentation	Wistar rats	[35]
Copper	Induces apoptosis, damages DNA, and causes poor sperm quality	Humans	[39]
	Decreases motility and lowers testosterone levels and sperm concentration	Nile tilapia fish (*Oreochromis niloticus*)	[40]

## Data Availability

Not applicable.

## References

[1] Morgan R.N., Ali A.A., Alshahrani M.Y., Aboshanab K.M. (2023). New Insights on Biological Activities, Chemical Compositions, and Classifications of Marine Actinomycetes Antifouling Agents. Microorganisms.

[2] Bixler G.D., Bhushan B. (2012). Biofouling: Lessons from nature. Philos. Trans. Ser. A Math. Phys. Eng. Sci..

[3] Bazaka K., Jacob M.V., Crawford R.J., Ivanova E.P. (2012). Efficient surface modification of biomaterial to prevent biofilm formation and the attachment of microorganisms. Appl. Microbiol. Biotechnol..

[4] Mai H., Cachot J., Brune J., Geffard O., Belles A., Budzinski H., Morin B. (2012). Embryotoxic and genotoxic effects of heavy metals and pesticides on early life stages of Pacific oyster (*Crassostrea gigas*). Mar. Pollut. Bull..

[5] Wisniewski P., Romano R.M., Kizys M.M., Oliveira K.C., Kasamatsu T., Giannocco G., Chiamolera M.I., Dias-da-Silva M.R., Romano M.A. (2015). Adult exposure to bisphenol A (BPA) in Wistar rats reduces sperm quality with disruption of the hypothalamic-pituitary-testicular axis. Toxicology.

[6] Horie Y., Yamagishi T., Shintaku Y., Iguchi T., Tatarazako N. (2018). Effects of tributyltin on early life-stage, reproduction, and gonadal sex differentiation in Japanese medaka (*Oryzias latipes*). Chemosphere.

[7] Chen L., Ye R., Zhang W., Hu C., Zhou B., Peterson D.R., Au D.W., Lam P.K., Qian P.Y. (2016). Endocrine Disruption throughout the Hypothalamus-Pituitary-Gonadal-Liver (HPGL) Axis in Marine Medaka (*Oryzias melastigma*) Chronically Exposed to the Antifouling and Chemopreventive Agent, 3,3′-Diindolylmethane (DIM). Chem. Res. Toxicol..

[8] Noda T., Morita S., Yamano T., Shimizu M., Nakamura T., Saitoh M., Yamada A. (1991). Teratogenicity study of tri-n-butyltin acetate in rats by oral administration. Toxicol. Lett..

[9] Cao Z., Li P., Cao X., Wang X., Liu B., He S., Gao G., Lu R., Li Z.H. (2022). Reproductive toxicity of environmental levels of triphenyltin to the marine rotifer, *Brachionus plicatilis*. Comp. Biochem. Physiol. Toxicol. Pharmacol. CBP.

[10] Nogueira A.F., Pereira J.L., Antunes S.C., Gonçalves F.J.M., Nunes B. (2018). Effects of zinc pyrithione on biochemical parameters of the freshwater Asian clam Corbicula fluminea. Aquat. Toxicol..

[11] Krzastek S.C., Farhi J., Gray M., Smith R.P. (2020). Impact of environmental toxin exposure on male fertility potential. Transl. Androl. Urol..

[12] Xing J.S., Bai Z.M. (2018). Is testicular dysgenesis syndrome a genetic, endocrine, or environmental disease, or an unexplained reproductive disorder?. Life Sci..

[13] Delessard M., Saulnier J., Rives A., Dumont L., Rondanino C., Rives N. (2020). Exposure to Chemotherapy During Childhood or Adulthood and Consequences on Spermatogenesis and Male Fertility. Int. J. Mol. Sci..

[14] Giulioni C., Maurizi V., Castellani D., Scarcella S., Skrami E., Balercia G., Galosi A.B. (2022). The environmental and occupational influence of pesticides on male fertility: A systematic review of human studies. Andrology.

[15] Rato L., Sousa A.C.A. (2021). The Impact of Endocrine-Disrupting Chemicals in Male Fertility: Focus on the Action of Obesogens. J. Xenobiotics.

[16] Arzuaga X., Smith M.T., Gibbons C.F., Skakkebæk N.E., Yost E.E., Beverly B.E.J., Hotchkiss A.K., Hauser R., Pagani R.L., Schrader S.M. (2019). Proposed Key Characteristics of Male Reproductive Toxicants as an Approach for Organizing and Evaluating Mechanistic Evidence in Human Health Hazard Assessments. Environ. Health Perspect..

[17] Skinner M.K. (2014). Endocrine disruptor induction of epigenetic transgenerational inheritance of disease. Mol. Cell. Endocrinol..

[18] Nayak J., Jena S.R., Kumar S., Kar S., Dixit A., Samanta L. (2023). Human sperm proteome reveals the effect of environmental borne seminal polyaromatic hydrocarbons exposome in etiology of idiopathic male factor infertility. Front. Cell Dev. Biol..

[19] Chianese R., Troisi J., Richards S., Scafuro M., Fasano S., Guida M., Pierantoni R., Meccariello R. (2018). Bisphenol A in Reproduction: Epigenetic Effects. Curr. Med. Chem..

[20] Yousef M.I., Kamel K.I., Hassan M.S., El-Morsy A.M. (2010). Protective role of propolis against reproductive toxicity of triphenyltin in male rabbits. Food Chem. Toxicol. Int. J. Publ. Br. Ind. Biol. Res. Assoc..

[21] De Castro T.F., Varela Junior A.S., Padilha F.F., Droppa-Almeida D., Saalfeld G.Q., Pires D.M., Pereira J.R., Corcini C.D., Colares E.P. (2019). Effects of exposure to triphenyltin (TPT) contaminant on sperm activity in adulthood of Calomys laucha exposed through breastfeeding. Environ. Sci. Pollut. Res. Int..

[22] Hou Y., Wang L.J., Jin Y.H., Guo R.Y., Yang L., Li E.C., Zhang J.L. (2022). Triphenyltin exposure induced abnormal morphological colouration in adult male guppies (*Poecilia reticulata*). Ecotoxicol. Environ. Saf..

[23] Horie Y., Watanabe H., Takanobu H., Shigemoto Y., Yamagishi T., Iguchi T., Tatarazako N. (2017). Effects of triphenyltin on reproduction in Japanese medaka (*Oryzias latipes*) across two generations. Aquat. Toxicol..

[24] Lan X.R., Li Y.W., Chen Q.L., Shen Y.J., Liu Z.H. (2020). Tributyltin impaired spermatogenesis and reproductive behavior in male zebrafish. Aquat. Toxicol..

[25] Xiao W.Y., Li Y.W., Chen Q.L., Liu Z.H. (2018). Tributyltin impaired reproductive success in female zebrafish through disrupting oogenesis, reproductive behaviors and serotonin synthesis. Aquat. Toxicol..

[26] Chabchoub I., Nouioui M.A., Araoud M., Mabrouk M., Amira D., Ben Aribia M.H., Mahmoud K., Zhioua F., Merdassi G., Hedhili A. (2021). Effects of lead, cadmium, copper and zinc levels on the male reproductive function. Andrologia.

[27] Pinto G.L., da Silva Castro J., Val A.L. (2021). Copper and cadmium impair sperm performance, fertilization and hatching of oocytes from Amazonian fish *Colossoma macropomum*. Chemosphere.

[28] Bhardwaj J.K., Paliwal A., Saraf P. (2021). Effects of heavy metals on reproduction owing to infertility. J. Biochem. Mol. Toxicol..

[29] Akinloye O., Arowojolu A.O., Shittu O.B., Anetor J.I. (2006). Cadmium toxicity: A possible cause of male infertility in Nigeria. Reprod. Biol..

[30] Bhardwaj J.K., Panchal H., Saraf P. (2021). Cadmium as a testicular toxicant: A Review. J. Appl. Toxicol. JAT.

[31] Xiong L., Zhou B., Liu H., Cai L. (2021). Comprehensive Review of Cadmium Toxicity Mechanisms in Male Reproduction and Therapeutic Strategies. Rev. Environ. Contam. Toxicol..

[32] Yi L., Dai J., Chen Y., Tong Y., Li Y., Fu G., Teng Z., Huang J., Quan C., Zhang Z. (2021). Reproductive toxicity of cadmium in pubertal male rats induced by cell apoptosis. Toxicol. Ind. Health.

[33] Yi L., Shang X.J., Lv L., Wang Y., Zhang J., Quan C., Shi Y., Liu Y., Zhang L. (2022). Cadmium-induced apoptosis of Leydig cells is mediated by excessive mitochondrial fission and inhibition of mitophagy. Cell Death Dis..

[34] Iqbal T., Cao M., Zhao Z., Zhao Y., Chen L., Chen T., Li C., Zhou X. (2021). Damage to the Testicular Structure of Rats by Acute Oral Exposure of Cadmium. Int. J. Environ. Res. Public Health.

[35] Cupertino M.C., Novaes R.D., Santos E.C., Neves A.C., Silva E., Oliveira J.A., Matta S.L.P. (2017). Differential Susceptibility of Germ and Leydig Cells to Cadmium-Mediated Toxicity: Impact on Testis Structure, Adiponectin Levels, and Steroidogenesis. Oxidative Med. Cell. Longev..

[36] Li X., Ru S., Tian H., Zhang S., Lin Z., Gao M., Wang J. (2021). Combined exposure to environmentally relevant copper and 2,2′-dithiobis-pyridine induces significant reproductive toxicity in male guppy (*Poecilia reticulata*). Sci. Total Environ..

[37] Zou L., Cheng G., Xu C., Liu H., Wang Y., Li N., Fan X., Zhu C., Xia W. (2021). Copper Nanoparticles Induce Oxidative Stress via the Heme Oxygenase 1 Signaling Pathway in vitro Studies. Int. J. Nanomed..

[38] Chen H., Wang Y., Luo J., Kang M., Hou J., Tang R., Zhao L., Shi F., Ye G., He X. (2022). Autophagy and apoptosis mediated nano-copper-induced testicular damage. Ecotoxicol. Environ. Saf..

[39] Wang Y.X., Wang P., Feng W., Liu C., Yang P., Chen Y.J., Sun L., Sun Y., Yue J., Gu L.J. (2017). Relationships between seminal plasma metals/metalloids and semen quality, sperm apoptosis and DNA integrity. Environ. Pollut..

[40] Oda S.S., El-Manakhly E.M., Abou-Srag M.A., Tohamy H.G. (2022). Assessment of reproductive toxicity of carbofuran and copper sulfate in male Nile tilapia (*Oreochromis niloticus*). Environ. Sci. Pollut. Res. Int..

[41] Fırat Ö., Erol R., Fırat Ö. (2022). Effects of Individual and Co-exposure of Copper Oxide Nanoparticles and Copper Sulphate on Nile Tilapia *Oreochromis niloticus*: Nanoparticles Enhance Pesticide Biochemical Toxicity. Acta Chim. Slov..

[42] Oriakpono O., Okorie D. (2022). Reproductive toxicity and biomaker response of male albino rats (*Rattus norvegicus*) exposed to cuprous oxide and petrol. GSC Biol. Pharm. Sci..

[43] Yang M., Hu J., Xia M., Wang Y., Tian F., Li W., Sun Y., Zhou Z. (2019). Zinc pyrithione induces immobilization of human spermatozoa and suppresses the response of the cAMP/PKA signaling pathway. Eur. J. Pharm. Sci. Off. J. Eur. Fed. Pharm. Sci..

[44] Nunes B., Costa M. (2019). Study of the effects of zinc pyrithione in biochemical parameters of the Polychaeta Hediste diversicolor: Evidences of neurotoxicity at ecologically relevant concentrations. Environ. Sci. Pollut. Res. Int..

[45] Shin D., Choi Y., Soon Z.Y., Kim M., Kim D.J., Jung J.H. (2022). Comparative toxicity study of waterborne two booster biocides (CuPT and ZnPT) on embryonic flounder (*Paralichthys olivaceus*). Ecotoxicol. Environ. Saf..

[46] Schwartz J.R. (2016). Zinc Pyrithione: A Topical Antimicrobial with Complex Pharmaceutics. J. Drugs Dermatol. JDD.

[47] Mangion S.E., Holmes A.M., Roberts M.S. (2021). Targeted Delivery of Zinc Pyrithione to Skin Epithelia. Int. J. Mol. Sci..

[48] Reeder N.L., Xu J., Youngquist R.S., Schwartz J.R., Rust R.C., Saunders C.W. (2011). The antifungal mechanism of action of zinc pyrithione. Br. J. Dermatol..

[49] Kumari N., Bhattacharya S.N., Das S., Datt S., Singh T., Jassal M., Agrawal A.K. (2021). In Situ Functionalization of Cellulose with Zinc Pyrithione for Antimicrobial Applications. ACS Appl. Mater. Interfaces.

[50] Mangion S.E., Sandiford L., Mohammed Y., Roberts M.S., Holmes A.M. (2022). Multi-Modal Imaging to Assess the Follicular Delivery of Zinc Pyrithione. Pharmaceutics.

[51] Thomas K.V. (1999). Determination of the antifouling agent zinc pyrithione in water samples by copper chelate formation and high-performance liquid chromatography-atmospheric pressure chemical ionisation mass spectrometry. J. Chromatogr. A.

[52] Onduka T., Mochida K., Harino H., Ito K., Kakuno A., Fujii K. (2010). Toxicity of metal pyrithione photodegradation products to marine organisms with indirect evidence for their presence in seawater. Arch. Environ. Contam. Toxicol..

[53] Zhao Y., Liu Y., Sun J., Sha H., Yang Y., Ye Q., Yang Q., Huang B., Yu Y., Huang H. (2018). Acute toxic responses of embryo-larval zebrafish to zinc pyrithione (ZPT) reveal embryological and developmental toxicity. Chemosphere.

[54] Bellas J. (2005). Toxicity assessment of the antifouling compound zinc pyrithione using early developmental stages of the ascidian Ciona intestinalis. Biofouling.

[55] Maraldo K., Dahllöf I. (2004). Indirect estimation of degradation time for zinc pyrithione and copper pyrithione in seawater. Mar. Pollut. Bull..

[56] Nunes B., Braga M.R., Campos J.C., Gomes R., Ramos A.S., Antunes S.C., Correia A.T. (2015). Ecotoxicological effect of zinc pyrithione in the freshwater fish *Gambusia holbrooki*. Ecotoxicology.

[57] Marcheselli M., Conzo F., Mauri M., Simonini R. (2010). Novel antifouling agent–zinc pyrithione: Short- and long-term effects on survival and reproduction of the marine polychaete *Dinophilus gyrociliatus*. Aquat. Toxicol..

[58] Sousa A.P., Nunes B. (2020). Standard and biochemical toxicological effects of zinc pyrithione in Daphnia magna and Daphnia longispina. Environ. Toxicol. Pharmacol..

[59] Cima F., Ballarin L. (2015). Immunotoxicity in ascidians: Antifouling compounds alternative to organotins-IV. The case of zinc pyrithione. Comp. Biochem. Physiol. Toxicol. Pharmacol. CBP.

[60] Holmes A.M., Kempson I., Turnbull T., Paterson D., Roberts M.S. (2018). Imaging the penetration and distribution of zinc and zinc species after topical application of zinc pyrithione to human skin. Toxicol. Appl. Pharmacol..

[61] Ren T., Fu G.H., Liu T.F., Hu K., Li H.R., Fang W.H., Yang X.L. (2017). Toxicity and accumulation of zinc pyrithione in the liver and kidneys of *Carassius auratus* gibelio: Association with P-glycoprotein expression. Fish Physiol. Biochem..

[62] Agnihotri S.K., Agrawal A.K., Hakim B.A., Vishwakarma A.L., Narender T., Sachan R., Sachdev M. (2016). Mitochondrial membrane potential (MMP) regulates sperm motility. Vitr. Cell. Dev. Biol. Anim..

[63] Jin H., Ma T., Sha X., Liu Z., Zhou Y., Meng X., Chen Y., Han X., Ding J. (2021). Polystyrene microplastics induced male reproductive toxicity in mice. J. Hazard. Mater..

[64] Grande G., Barrachina F., Soler-Ventura A., Jodar M., Mancini F., Marana R., Chiloiro S., Pontecorvi A., Oliva R., Milardi D. (2022). The Role of Testosterone in Spermatogenesis: Lessons from Proteome Profiling of Human Spermatozoa in Testosterone Deficiency. Front. Endocrinol..

[65] Hu J., Luo X., Panga M.J., Appiah C., Retyunskiy V., Zhu L., Zhao Y. (2024). Toxic effects and potential mechanisms of zinc pyrithione (ZPT) exposure on sperm and testicular injury in zebrafish. J. Hazard. Mater..

[66] Lu M., Mu Y., Liu Y. (2022). Triphenyltin disrupts the testicular microenvironment and reduces sperm quality in adult male rats. Chemosphere.

[67] Ge F., Zheng W., Bao S., Wu K., Xiang S., Chen W., Chen X., Mo J., Zhou S., Wang Y. (2018). In utero exposure to triphenyltin disrupts rat fetal testis development. Chemosphere.

[68] Ghaemmaleki F., Mohammadi P., Baeeri M., Navaei-Nigjeh M., Abdollahi M., Mostafalou S. (2020). Estrogens counteract tributyltin-induced toxicity in the rat islets of Langerhans. Heliyon.

[69] Antizar-Ladislao B. (2008). Environmental levels, toxicity and human exposure to tributyltin (TBT)-contaminated marine environment. A review. Environ. Int..

[70] Mitra S., Srivastava A., Khandelwal S. (2017). Long term impact of the endocrine disruptor tributyltin on male fertility following a single acute exposure. Environ. Toxicol..

[71] Rani K.U., Musthafa M.S., War M., Al-Sadoon M.K., Paray B.A., Shareef T.H., Nawas P.M. (2015). Impact of tributyltin on antioxidant and DNA damage response in spermatozoa of freshwater prawn Macrobrachium rosenbergii. Environ. Sci. Pollut. Res. Int..

[72] Revathi P., Iyapparaj P., Vasanthi L.A., Munuswamy N., Prasanna V.A., Pandiyarajan J., Krishnan M. (2014). Influence of short term exposure of TBT on the male reproductive activity in freshwater prawn Macrobrachium rosenbergii (De Man). Bull. Environ. Contam. Toxicol..

[73] Revathi P., Iyapparaj P., Vasanthi L.A., Munuswamy N., Krishnan M. (2014). Ultrastructural changes during spermatogenesis, biochemical and hormonal evidences of testicular toxicity caused by TBT in freshwater prawn *Macrobrachium rosenbergii* (De Man, 1879). Environ. Toxicol..

[74] Takalani N.B., Monageng E.M., Mohlala K., Monsees T.K., Henkel R., Opuwari C.S. (2023). Role of oxidative stress in male infertility. Reprod. Fertil..

[75] Beygi Z., Forouhari S., Mahmoudi E., Hayat S.M.G., Nourimand F. (2021). Role of Oxidative Stress and Antioxidant Supplementation in Male Fertility. Curr. Mol. Med..

[76] Barati E., Nikzad H., Karimian M. (2020). Oxidative stress and male infertility: Current knowledge of pathophysiology and role of antioxidant therapy in disease management. Cell. Mol. Life Sci. CMLS.

[77] Juárez-Rojas L., Casillas F., López A., Betancourt M., Ommati M.M., Retana-Márquez S. (2022). Physiological role of reactive oxygen species in testis and epididymal spermatozoa. Andrologia.

[78] Du Plessis S.S., Agarwal A., Halabi J., Tvrda E. (2015). Contemporary evidence on the physiological role of reactive oxygen species in human sperm function. J. Assist. Reprod. Genet..

[79] Le Gal K., Schmidt E.E., Sayin V.I. (2021). Cellular Redox Homeostasis. Antioxidants.

[80] Sies H., Berndt C., Jones D.P. (2017). Oxidative Stress. Annu. Rev. Biochem..

[81] De Almeida A., de Oliveira J., da Silva Pontes L.V., de Souza Júnior J.F., Gonçalves T.A.F., Dantas S.H., de Almeida Feitosa M.S., Silva A.O., de Medeiros I.A. (2022). ROS: Basic Concepts, Sources, Cellular Signaling, and its Implications in Aging Pathways. Oxidative Med. Cell. Longev..

[82] Cito G., Becatti M., Natali A., Fucci R., Picone R., Cocci A., Falcone P., Criscuoli L., Mannucci A., Argento F.R. (2020). Redox status assessment in infertile patients with non-obstructive azoospermia undergoing testicular sperm extraction: A prospective study. Andrology.

[83] Agarwal A., Roychoudhury S., Sharma R., Gupta S., Majzoub A., Sabanegh E. (2017). Diagnostic application of oxidation-reduction potential assay for measurement of oxidative stress: Clinical utility in male factor infertility. Reprod. Biomed. Online.

[84] Mannucci A., Argento F.R., Fini E., Coccia M.E., Taddei N., Becatti M., Fiorillo C. (2021). The Impact of Oxidative Stress in Male Infertility. Front. Mol. Biosci..

[85] Lennicke C., Cochemé H.M. (2021). Redox metabolism: ROS as specific molecular regulators of cell signaling and function. Mol. Cell.

[86] Wagner H., Cheng J.W., Ko E.Y. (2018). Role of reactive oxygen species in male infertility: An updated review of literature. Arab J. Urol..

[87] Ighodaro O.M., Akinloye O.A. (2018). First line defence antioxidants-superoxide dismutase (SOD), catalase (CAT) and glutathione peroxidase (GPX): Their fundamental role in the entire antioxidant defence grid. Alex. J. Med..

[88] Marklund S.L. (1984). Extracellular superoxide dismutase and other superoxide dismutase isoenzymes in tissues from nine mammalian species. Biochem. J..

[89] Góth L., Rass P., Páy A. (2004). Catalase enzyme mutations and their association with diseases. Mol. Diagn. A J. Devoted Underst. Hum. Dis. Through Clin. Appl. Mol. Biol..

[90] Bui A.D., Sharma R., Henkel R., Agarwal A. (2018). Reactive oxygen species impact on sperm DNA and its role in male infertility. Andrologia.

[91] Ribas-Maynou J., Yeste M. (2020). Oxidative Stress in Male Infertility: Causes, Effects in Assisted Reproductive Techniques, and Protective Support of Antioxidants. Biology.

[92] González-Marín C., Gosálvez J., Roy R. (2012). Types, causes, detection and repair of DNA fragmentation in animal and human sperm cells. Int. J. Mol. Sci..

[93] Dutta S., Majzoub A., Agarwal A. (2019). Oxidative stress and sperm function: A systematic review on evaluation and management. Arab J. Urol..

[94] Chianese R., Pierantoni R. (2021). Mitochondrial Reactive Oxygen Species (ROS) Production Alters Sperm Quality. Antioxidants.

[95] Qiu Y., Yang H., Li C., Xu C. (2020). Progress in Research on Sperm DNA Fragmentation. Med. Sci. Monit. Int. Med. J. Exp. Clin. Res..

[96] Min B.H., Saravanan M., Nam S.E., Eom H.J., Rhee J.S. (2019). Waterborne zinc pyrithione modulates immunity, biochemical, and antioxidant parameters in the blood of olive flounder. Fish Shellfish Immunol..

[97] Wu K., Li Y., Liu J., Mo J., Li X., Ge R.S. (2020). Long-term triphenyltin exposure disrupts adrenal function in adult male rats. Chemosphere.

[98] Ishihara Y., Kawami T., Ishida A., Yamazaki T. (2012). Tributyltin induces oxidative stress and neuronal injury by inhibiting glutathione S-transferase in rat organotypic hippocampal slice cultures. Neurochem. Int..

[99] Hosen M.B., Islam M.R., Begum F., Kabir Y., Howlader M.Z. (2015). Oxidative stress induced sperm DNA damage, a possible reason for male infertility. Iran. J. Reprod. Med..

[100] Rashki Ghaleno L., Alizadeh A., Drevet J.R., Shahverdi A., Valojerdi M.R. (2021). Oxidation of Sperm DNA and Male Infertility. Antioxidants.

[101] Iommiello V.M., Albani E., Di Rosa A., Marras A., Menduni F., Morreale G., Levi S.L., Pisano B., Levi-Setti P.E. (2015). Ejaculate oxidative stress is related with sperm DNA fragmentation and round cells. Int. J. Endocrinol..

[102] Aitken R.J., Smith T.B., Jobling M.S., Baker M.A., De Iuliis G.N. (2014). Oxidative stress and male reproductive health. Asian J. Androl..

[103] Nowicka-Bauer K., Lepczynski A., Ozgo M., Kamieniczna M., Fraczek M., Stanski L., Olszewska M., Malcher A., Skrzypczak W., Kurpisz M.K. (2018). Sperm mitochondrial dysfunction and oxidative stress as possible reasons for isolated asthenozoospermia. J. Physiol. Pharmacol. Off. J. Pol. Physiol. Soc..

[104] Piomboni P., Focarelli R., Stendardi A., Ferramosca A., Zara V. (2012). The role of mitochondria in energy production for human sperm motility. Int. J. Androl..

[105] La Vignera S., Condorelli R., Vicari E., D’Agata R., Calogero A.E. (2012). Diabetes mellitus and sperm parameters. J. Androl..

[106] Daigneault B.W., de Agostini Losano J.D. (2022). Tributyltin chloride exposure to post-ejaculatory sperm reduces motility, mitochondrial function and subsequent embryo development. Reprod. Fertil. Dev..

[107] Chen M., Ding Y., Ke Y., Zeng Y., Liu N., Zhong Y., Hua X., Li Z., Xiong Y., Wu C. (2020). Anti-tumour activity of zinc ionophore pyrithione in human ovarian cancer cells through inhibition of proliferation and migration and promotion of lysosome-mitochondrial apoptosis. Artif. Cells Nanomed. Biotechnol..

[108] Mo J., Lin D., Wang J., Li P., Liu W. (2018). Apoptosis in HepG2 cells induced by zinc pyrithione via mitochondrial dysfunction pathway: Involvement of zinc accumulation and oxidative stress. Ecotoxicol. Environ. Saf..

[109] Mann J.J., Fraker P.J. (2005). Zinc pyrithione induces apoptosis and increases expression of Bim. Apoptosis Int. J. Program. Cell Death.

[110] Aitken R.J., Drevet J.R. (2020). The Importance of Oxidative Stress in Determining the Functionality of Mammalian Spermatozoa: A Two-Edged Sword. Antioxidants.

[111] Görlach A., Bertram K., Hudecova S., Krizanova O. (2015). Calcium and ROS: A mutual interplay. Redox Biol..

[112] Plaisance V., Brajkovic S., Tenenbaum M., Favre D., Ezanno H., Bonnefond A., Bonner C., Gmyr V., Kerr-Conte J., Gauthier B.R. (2016). Endoplasmic Reticulum Stress Links Oxidative Stress to Impaired Pancreatic Beta-Cell Function Caused by Human Oxidized LDL. PLoS ONE.

[113] Zhao Y., Wang H., Duah P.A., Retyunskiy V., Liu Y., Chen G. (2022). Zinc pyrithione (ZPT) -induced embryonic toxicogenomic responses reveal involvement of oxidative damage, apoptosis, endoplasmic reticulum (ER) stress and autophagy. Aquat. Toxicol..

[114] Don Wai Luu L., Kaakoush N.O., Castaño-Rodríguez N. (2022). The role of ATG16L2 in autophagy and disease. Autophagy.

[115] Kruk J., Aboul-Enein H.Y., Kładna A., Bowser J.E. (2019). Oxidative stress in biological systems and its relation with pathophysiological functions: The effect of physical activity on cellular redox homeostasis. Free Radic. Res..

[116] Aitken R.J. (2017). Reactive oxygen species as mediators of sperm capacitation and pathological damage. Mol. Reprod. Dev..

[117] Aitken R.J., Baker M.A., Nixon B. (2015). Are sperm capacitation and apoptosis the opposite ends of a continuum driven by oxidative stress?. Asian J. Androl..

[118] Tang L., Liu Y.L., Qin G., Lin Q., Zhang Y.H. (2021). Effects of tributyltin on gonad and brood pouch development of male pregnant lined seahorse (*Hippocampus erectus*) at environmentally relevant concentrations. J. Hazard. Mater..

[119] Haque M.N., Nam S.E., Eom H.J., Kim S.K., Rhee J.S. (2020). Exposure to sublethal concentrations of zinc pyrithione inhibits growth and survival of marine polychaete through induction of oxidative stress and DNA damage. Mar. Pollut. Bull..

[120] Bodiga V.L., Vemuri P.K., Nimmagadda G., Bodiga S. (2020). Zinc-dependent changes in oxidative and endoplasmic reticulum stress during cardiomyocyte hypoxia/reoxygenation. Biol. Chem..

[121] Cenini G., Lloret A., Cascella R. (2020). Oxidative Stress and Mitochondrial Damage in Neurodegenerative Diseases: From Molecular Mechanisms to Targeted Therapies. Oxidative Med. Cell. Longev..

[122] Demine S., Renard P., Arnould T. (2019). Mitochondrial Uncoupling: A Key Controller of Biological Processes in Physiology and Diseases. Cells.

[123] Ma H., Yan X., Yan L., Zhao J., Song J., Peng R., Yang Y., Peng J., Liu K. (2021). Identification and Functional Analysis of Apoptotic Protease Activating Factor-1 (Apaf-1) from *Spodoptera litura*. Insects.

[124] Jäger R., Bertrand M.J., Gorman A.M., Vandenabeele P., Samali A. (2012). The unfolded protein response at the crossroads of cellular life and death during endoplasmic reticulum stress. Biol. Cell.

[125] Mizrahi R., Breitbart H. (2014). Mitochondrial PKA mediates sperm motility. Biochim. Et Biophys. Acta.

[126] Legoff L., D’Cruz S.C., Lebosq M., Gely-Pernot A., Bouchekhchoukha K., Monfort C., Kernanec P.Y., Tevosian S., Multigner L., Smagulova F. (2021). Developmental exposure to chlordecone induces transgenerational effects in somatic prostate tissue which are associated with epigenetic histone trimethylation changes. Environ. Int..

[127] Akbari S., Amiri F.T., Naderi M., Shaki F., Seyedabadi M. (2022). Sodium arsenite accelerates D-galactose-induced aging in the testis of the rat: Evidence for mitochondrial oxidative damage, NF-kB, JNK, and apoptosis pathways. Toxicology.

[128] Hao L., Ru S., Qin J., Wang W., Zhang J., Wei S., Wang J., Zhang X. (2022). Transgenerational effects of parental bisphenol S exposure on zebrafish (*Danio rerio*) reproduction. Food Chem. Toxicol. Int. J. Publ. Br. Ind. Biol. Res. Assoc..

[129] Dorus S., Skerget S., Karr T.L. (2012). Proteomic discovery of diverse immunity molecules in mammalian spermatozoa. Syst. Biol. Reprod. Med..

[130] Cannarella R., Crafa A., Barbagallo F., Mongioì L.M., Condorelli R.A., Aversa A., Calogero A.E., La Vignera S. (2020). Seminal Plasma Proteomic Biomarkers of Oxidative Stress. Int. J. Mol. Sci..

[131] Sahu C., Singla S., Jena G. (2022). Studies on male gonadal toxicity of bisphenol A in diabetic rats: An example of exacerbation effect. J. Biochem. Mol. Toxicol..

[132] Caldarola G., Milardi D., Grande G., Quercia A., Baroni S., Morelli R., Marana R., Pontecorvi A., De Simone C., Peris K. (2017). Untreated Psoriasis Impairs Male Fertility: A Case-Control Study. Dermatology.

[133] Zhang H., Ren C., Liu Q., Wang Q., Wang D. (2023). TFAP2C exacerbates psoriasis-like inflammation by promoting Th17 and Th1 cells activation through regulating TEAD4 transcription. Allergol. Et Immunopathol..

[134] Celik-Ozenci C., Tasatargil A. (2013). Role of poly(ADP-ribose) polymerases in male reproduction. Spermatogenesis.

[135] Hanis T., Zidek V., Sachova J., Klir P., Deyl Z. (1989). Effects of dietary trans-fatty acids on reproductive performance of Wistar rats. Br. J. Nutr..

[136] Veaute C., Andreoli M.F., Racca A., Bailat A., Scalerandi M.V., Bernal C., Malan Borel I. (2007). Effects of isomeric fatty acids on reproductive parameters in mice. Am. J. Reprod. Immunol..

